# Humoral Response in Hemodialysis Patients Post-SARS-CoV-2 mRNA Vaccination: A Systematic Review of Literature

**DOI:** 10.3390/vaccines11040724

**Published:** 2023-03-24

**Authors:** Kin Israel Notarte, Jesus Alfonso Catahay, Princess Juneire Peligro, Jacqueline Veronica Velasco, Abbygail Therese Ver, Jonathan Jaime Guerrero, Jin Liu, Giuseppe Lippi, Stefanie W. Benoit, Brandon Michael Henry, César Fernández-de-las-Peñas

**Affiliations:** 1 Department of Pathology, Johns Hopkins University School of Medicine, Baltimore, MD 21205, USA; kinotarte@gmail.com (K.I.N.); jliu221@jhmi.edu (J.L.); 2 Department of Medicine, Saint Peter’s University Hospital, New Brunswick, NJ 08901, USA; jcatahay@saintpetersuh.com; 3 Faculty of Medicine and Surgery, University of Santo Tomas, Manila 1008, Philippines; princessjuneire.peligro.med@ust.edu.ph (P.J.P.); jacquelineveronica.velasco.med@ust.edu.ph (J.V.V.); abbygailtherese.ver.med@ust.edu.ph (A.T.V.); 4 Learning Unit IV, College of Medicine, University of the Philippines Manila, Manila 1001, Philippines; jonathanjaime.g.guerrero@gmail.com; 5 Section of Clinical Biochemistry, University of Verona, 37129 Verona, Italy; giuseppe.lippi@univr.it; 6 Clinical Laboratory, Division of Nephrology and Hypertension, Cincinnati Children’s Hospital Medical Center, Cincinnati, OH 45229, USA; stefanie.benoit@cchmc.org (S.W.B.); brandon.henry@cchmc.org (B.M.H.); 7 Department of Pediatrics, University of Cincinnati College of Medicine, Cincinnati, OH 45103, USA; 8 Department of Physical Therapy, Occupational Therapy, Physical Medicine and Rehabilitation, Universidad Rey Juan Carlos, 28922 Madrid, Spain

**Keywords:** COVID-19, mRNA-vaccines, hemodialysis, antibody titer, adaptive immunity

## Abstract

Severe Acute Respiratory Syndrome Coronavirus 2 (SARS-CoV-2), the causative agent of coronavirus disease 2019 (COVID-19), has infected over 600 million individuals and caused nearly 7 million deaths worldwide (10 January 2023). Patients with renal disease undergoing hemodialysis are among those most adversely affected, with an increased predisposition to SARS-CoV-2 infection and death. This systematic review aimed to pool evidence assessing the humoral response of hemodialysis patients (HDP) post-mRNA SARS-CoV-2 vaccination. A systematic search of the literature was performed through MEDLINE, CINAHL, PubMed, EMBASE, and Web of Science databases, as well as medRxiv and bioRxiv preprint servers up to 10 January 2023. Cohort and case-control studies were included if they reported an immune response in one group of patients undergoing hemodialysis who received mRNA SARS-CoV-2 vaccination compared with another group of patients receiving the same vaccine but not on hemodialysis. The methodological quality was assessed using the Newcastle-Ottawa Scale. Meta-analysis was not deemed appropriate due to the high heterogeneity between studies. From the 120 studies identified, nine (n = 1969 participants) met the inclusion criteria. Most studies (n = 8/9, 88%) were of high or medium methodological quality (≥6/9 stars). The results revealed that HDP developed lower antibody levels across all timepoints post-vaccination when compared with controls. Patients with chronic kidney disease elicited the highest antibody immune response, followed by HDP and, lastly, kidney transplant recipients. Overall, post-vaccination antibody titers were comparatively lower than in the healthy population. Current results imply that robust vaccination strategies are needed to address waning immune responses in vulnerable populations.

## 1. Introduction

Severe Acute Respiratory Syndrome Coronavirus 2 (SARS-CoV-2), the causative agent of coronavirus disease 2019 (COVID-19), has infected over 600 million individuals and caused nearly 7 million deaths worldwide (10 January 2023) [1]. Patients with end-stage renal disease who are undergoing hemodialysis are among those most adversely affected, with an increased predisposition to SARS-CoV-2 infection and death compared with the general population [2]. Alfano et al. stipulated that up to 37% of hemodialysis patients are at high risk of acquiring a SARS-CoV-2 infection, with a hospitalization rate up to 88% and a case-fatality rate over 20% [3]. These data are markedly higher than the reported rates in the general population, further emphasizing the need for early administration of COVID-19 vaccines to vulnerable groups [4]. However, previous studies have reported waning of anti-SARS-CoV-2 antibody titers over time following COVID-19 vaccination, with a rate of decay dependent upon host factors such as age, sex, serostatus, comorbidities, and treatments [5,6]. Hemodialysis patients have a diminished humoral response to the COVID-19 vaccination compared with the general population [2]. Hemodialysis patients who succumbed to natural SARS-CoV-2 infection have also shown declining antibody titers 3 months post-infection, thus raising the possibility of hypo-responsiveness to vaccination in this population [7]. Further, randomized clinical trials for the BNT162b2 vaccine had included a few patients with kidney disease [8]. Thus, there are limited data on the clinical efficacy of COVID-19 vaccination in preventing infection and adverse outcomes in hemodialysis patients [9]. Accordingly, this systematic review aimed at pooling clinical evidence to assess the humoral response of hemodialysis patients after receiving the mRNA vaccine.

## 2. Methods

This systematic literature review aims to assess the humoral response of hemodialysis patients post-mRNA SARS-CoV-2 vaccination following the Preferred Reporting Items for Systematic Reviews and Meta-Analyses (PRISMA) statement of 2020 [10]. The review study was prospectively registered in the Open Science Framework (OSF) database at the following link: https://osf.io/6dku3.

### 2.1. Search Strategy and Selection Criteria

Two different authors conducted a search for articles published up to 10 January 2023 using the databases MEDLINE, CINAHL, PubMed, EMBASE, and Web of Science, as well as the preprint servers medRxiv and bioRxiv. The search terms included “hemodialysis” AND “humoral response” AND “COVID vaccine” OR “SARS-CoV-2 vaccine” OR “BNT162b2” OR “Pfizer-BioNTech” OR “mRNA-1273” OR “Moderna-NIAID”. Combinations of these terms using the Boolean operator were used for the search (Table 1).

The Population, Intervention, Comparison and Outcome (PICO) principle was used to describe the inclusion and exclusion criteria:

Population: Hemodialysis-dependent adults (>18 years) receiving COVID-19 vaccine

Intervention: Administration of one of these mRNA COVID-19 vaccines: BNT162b2

(“Pfizer-BioNTech”, Mainz, Germany), or mRNA-1273 (“Moderna-NIAID”).

Comparison: Adults (>18 years) who do not require hemodialysis receiving COVID-

19 vaccine.

Outcome: To measure antibody titer post-vaccine in adults undergoing hemodialysis

in comparison with adults not on hemodialysis.

### 2.2. Screening Process, Study Selection and Data Extraction

This systematic review included observational cohort, cross-sectional, and case-control studies where the response of a group of hemodialysis-dependent adults receiving any type of mRNA COVID-19 vaccine was compared with another group of adults receiving mRNA COVID-19 vaccine who do not require hemodialysis. Editorials, opinion, and correspondence articles without original data were excluded. The current review was limited to human studies and English language full-text papers.

Two authors screened the title and abstract of publications identified in the database search and removed all duplicates. The titles and abstracts were screened for eligibility, and those articles fulfilling inclusion criteria were fully read. The full text of eligible articles was retrieved and analyzed.

Data including authors, vaccine, country of origin, sample size, setting, median age, assay, and antibody titer were extracted from each study. The authors had to reach consensus on data extraction. Discrepancies between reviewers at any stage of the screening process were resolved in consultation with a third author.

### 2.3. Methodological Quality

The methodological quality of the studies was evaluated by two authors using the Newcastle-Ottawa Scale (NOS), a star rating system evaluating the risk of bias in case-control and cohort studies [11]. The NOS evaluates the following items in cohort studies: case selection (i.e., cohort, representativeness, selection of the non-exposed cohort, case definition, main outcome), comparability (i.e., between-groups comparison by controlling for age, gender, or other) and exposure (i.e., outcome assessment, duration of follow-up, adequate follow-up). Some items are adapted if a case-control study is evaluated. For instance, a case selection item includes adequate case definition or control group selection.

In cohort studies using a longitudinal design or case-control studies, a rating of 7 to 9 stars indicates high methodological quality, 5 to 6 stars indicates medium methodological quality, and less than or equal to 4 stars indicates low methodological quality. In cohort studies with a cross-sectional design, a maximum of 3 stars can be awarded. Studies scoring 3 stars are of good methodological quality, 2 stars are of fair methodological quality, and 1 star is of poor methodological quality. If there is disagreement, a third researcher arbitrates a consensus decision.

### 2.4. Data Synthesis

Meta-analysis was not deemed appropriate due to the high heterogeneity between studies. Therefore, we conducted a narrative synthesis of the data reported by addressing population, limitations, and methodological quality. We also reported the fold increase in Ab titer following primary or booster doses.

## 3. Results

### 3.1. Study Selection

**Figure 1:**
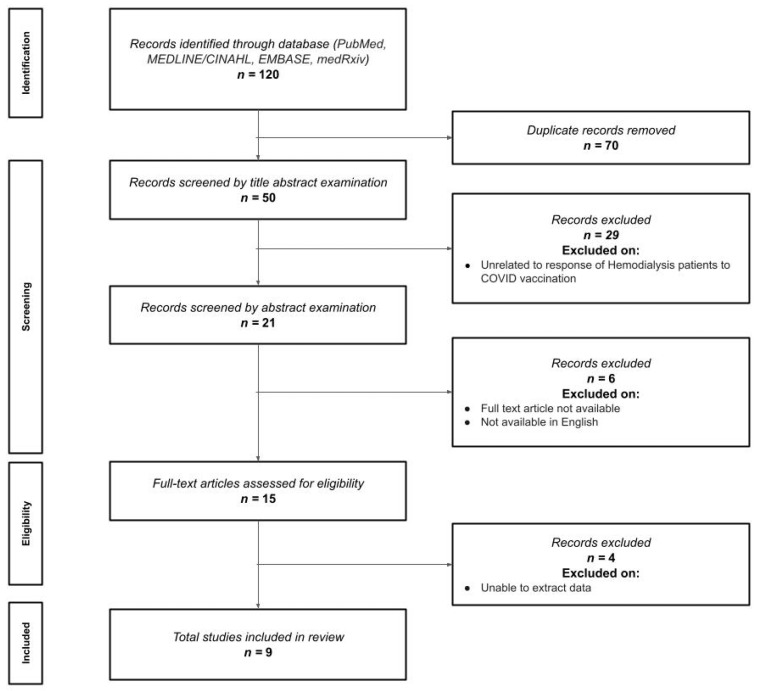
Preferred Reporting Items for Systematic Reviews and Meta-Analyses (PRISMA) Flow diagram.

The electronic searches identified 120 titles for initial screening. After removing duplicates (n = 70) and papers not related to post-vaccine changes in patients receiving hemodialysis (n = 29), 21 studies remained for abstract examination. Six were excluded after abstract examination, leading to a total of 15 papers for full-text review. Finally, nine articles [12,13,14,15,16,17,18,19,20] with a total population of 1969 participants were included (Figure 1).

### 3.2. Sample Characteristics

Three articles [12,15,16] were conducted in France, while the remaining were each from Portugal [17], Germany [18], Belgium [13], the Netherlands [14], the United States of America [20], and Austria [19]. Of the reviewed articles, the largest sample size (n = 800) was from the Netherlands [14].

Six articles [12,15,16,17,18,19] used BNT162b2 (Pfizer-BioNTech) vaccine (n = 485), two articles [14,20] mRNA-1273 (Moderna, Cambridge, MA, USA) vaccine (n = 863) and one [13] included both BNT162b2 (Pfizer-BioNTech) and mRNA-1273 (Moderna) vaccines (n = 618). Six studies administered two doses of the COVID-19 vaccine [12,13,14,15,17,19], while two studies administered three doses [16,20] to the participants. Just one study administered one dose [18]. Eight studies included participants who had no history of previous SARS-CoV-2 infection [12,13,14,15,16,17,18,19], while just one study included subjects who were previously infected by SARS-CoV-2 [20]. Three studies compared hemodialysis participants with a control group [12,13,14], two compared hemodialysis participants to other kidney pathologies, e.g., chronic kidney disease and kidney transplant recipients [14,15], and another two compared patients undergoing hemodialysis vs. peritoneal dialysis [16,17]. Table 2 summarizes the results of those studies investigating one or two doses, whereas Table 3 details the results of studies investigating changes after the third (booster) dose.

One study investigated the effect of age on hemodialysis participants [18]. One study compared the humoral response of hemodialysis patients and if seroconversion was present or not after a first vaccine dose [19], and another one compared the humoral response of hemodialysis patients infected with the wild-type strain vs. Omicron variant [20].

### 3.3. Methodological Quality

**Figure 2:**
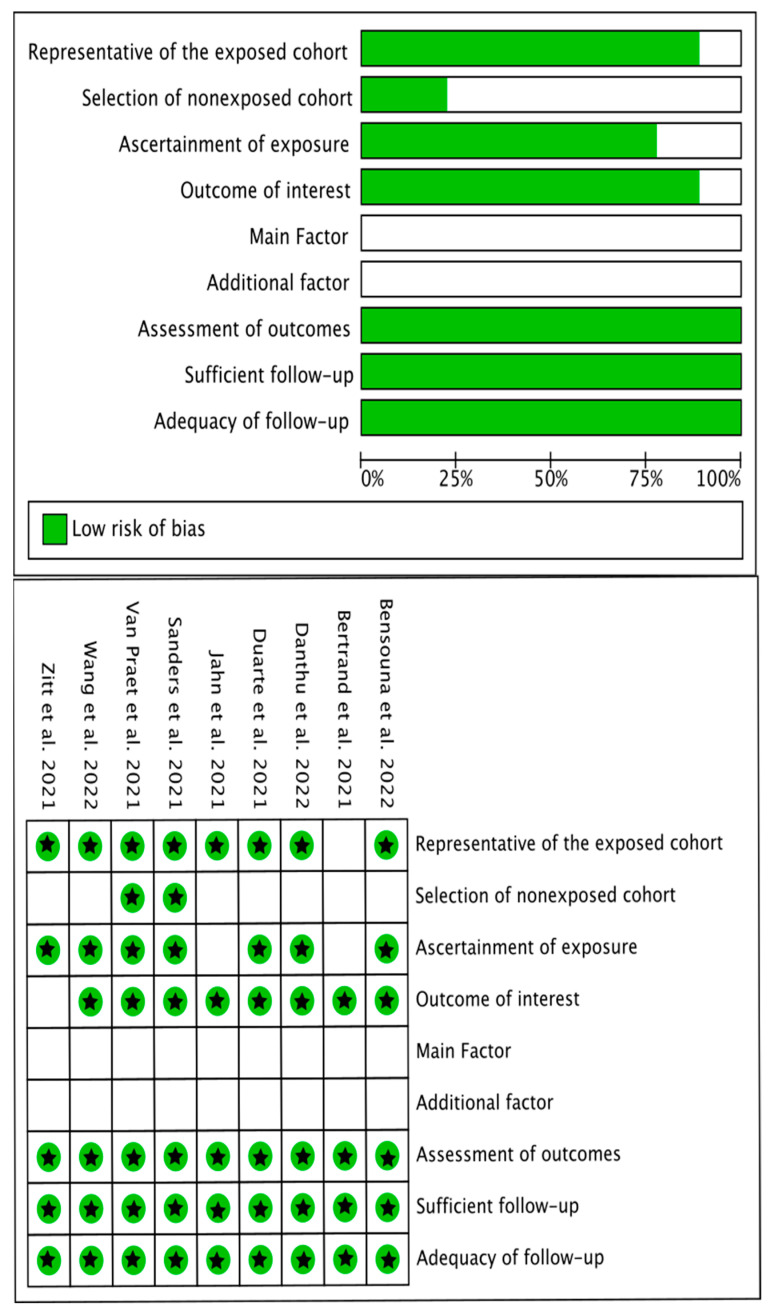
Methodological quality of those studies investigating humoral response in patients receiving hemodialysis after mRNA COVID-19 vaccine.

Two cohort studies were of high methodological quality (7/9 stars), six studies were of medium methodological quality (6/9 stars), and one was of low methodological quality (4/9 stars). No study matched the comparative group by age/gender or controlled for other factors (e.g., other comorbidities), thus none fulfilled these methodological criteria (Figure 2). No disagreement between authors was found. Table 4 presents the NOS score for each cohort study and a summary of every item.

### 3.4. Findings

Among studies that compared hemodialysis participants with control groups [12,13,14], hemodialysis participants developed significantly lower post-vaccine antibody levels across all timepoints. However, a consistent decline in the degree of disparity between both groups was observed as the days from the first vaccine dose administration increased.

Sanders et al. [14] found that patients undergoing hemodialysis had lower antibody levels compared with patients having chronic kidney disease but showed higher antibody levels compared with kidney transplant recipients after COVID-19 vaccination. A similar trend was observed by Bertrand et al. [15], wherein patients undergoing hemodialysis showed higher antibody levels compared with kidney transplant recipients after the second vaccine dose.

Bensouna et al. [16] reported no significant differences in antibody levels between patients undergoing hemodialysis and those undergoing peritoneal dialysis, whereas the study by Duarte et al. [17] observed higher antibody levels in those undergoing peritoneal dialysis.

Among patients undergoing hemodialysis, factors such as age, seroconversion after vaccination, the SARS-CoV-2 variant (wild-type strain vs. Omicron variant), and mRNA vaccine brand (Pfizer-BioNTech vs. Moderna-NIAID) and their association with antibody titer levels were assessed in three studies. Older individuals [18], those infected with the Omicron variant [20], and those vaccinated with Pfizer-BioNTech [13] developed lower antibody levels compared with their counterparts, i.e., younger subjects [18], those infected with the wild-type strain [20], and those vaccinated with the Moderna-NIAID vaccine [13].

## 4. Discussion

This systematic review explored the immune response of individuals undergoing hemodialysis after mRNA SARS-CoV-2 vaccination. Among the nine studies investigating hemodialysis patients, four [12,13,14,18] compared their results with controls. All studies presented consistent findings, showing that control groups had higher immune responses than hemodialysis patients. In other studies, hemodialysis patients were compared with those with other pathological conditions, such as chronic kidney disease or kidney transplant. Most studies were of high or medium methodological quality.

Overall, patients undergoing hemodialysis exhibit a hypo-responsiveness of the immune system after administration of one, two, or a booster dose of the mRNA SARS-CoV-2 vaccine. The results by Danthu et al. [12] suggested that immunosuppressive therapy may be a critical factor in the hypo-responsiveness of individuals undergoing hemodialysis. This is consistent with the analysis of risk factors for seroconversion failure conducted by Stumpf et al. [21], which stated that immunosuppressive therapy as well as vaccine type were potential risk factors for a negative seroconversion after mRNA COVID-19 vaccination. In fact, the immune response of patients undergoing hemodialysis was higher when more time elapsed from the first dose to the second or booster vaccine dose, suggesting that the immune system of these patients could need more time to develop antibodies due to their health status.

Focusing on differences in vaccine types, Van Praet et al. [13] compared immune responses from BNT162b2 (Pfizer-BioNTech) and mRNA-1273 (Moderna-NIAID) vaccines and found that Moderna-NIAID produced an overall higher immune response than Pfizer -BioNTech vaccine in individuals with hemodialysis. This is consistent with the results observed by Stumpf et al. [21], showing that the seroconversion success rate was higher after Moderna-NIAID than after to Pfizer-BioNTech COVID-19 vaccine. According to Van Praet el al. [13], the presence of a higher mRNA dose in Moderna-NIAID (100 μg) than in Pfizer-BioNTech’s (30 μg) vaccine is the most plausible explanation for the higher immune response seen in hemodialysis patients as well as in the general population.

In terms of age, results from two studies [16,18] showed that antibody responses are negatively correlated with older age, although these findings still need to be further investigated due to limitations such as the lack of control groups of older age. In fact, older age, male sex, seronegativity, and patients with comorbidities mounted less humoral immune responses [5].

Regarding different variants, Wang et al. [20] studied the neutralizing antibody (nAb) response of hemodialysis patients comparing Omicron and wild-type strains and found that the vaccinal (Moderna-NIAID) nAb response against the wild-type strain was significantly higher than in the Omicron variant after the third (booster) dose. This would be an expected finding since COVID-19 vaccines were originally developed based on the wild-type strain genome and not subsequent SARS-CoV-2 variants.

Additionally, we also observed a higher antibody titer response in patients with chronic kidney disease (CKD) stages 4 and 5 not receiving hemodialysis when compared with those receiving hemodialysis, albeit again, lower compared with the control population [14,15]. Studies have associated lower immune response in hemodialysis patients with a number of risk factors, including age older 65 [5,22,23,24], nonresponse to hepatitis B vaccination, low serum albumin, lymphocytosis, IgG levels, use of immunosuppressants, high dialysis inadequacy, dialysis vintage, and high intravenous iron dose [12,13,24,25,26,27]. Interestingly, when comparing hemodialysis and peritoneal dialysis patients, evidence shows mixed findings on whether there is a significant difference in the antibody titers between these groups [16,17]. Current evidence elaborates that peritoneal dialysis patients mount a greater response than hemodialysis patients [17,28,29], but still suboptimal compared with general population.

There was a considerable decrease in the immune response in post-kidney transplant recipients, thus indicating that immunosuppressive drugs are a more significant determinant of response to vaccination than hemodialysis [30]. Other factors which could contribute to a decreased immune response in kidney transplant recipients would include advanced age, the need for high doses of corticosteroids during the past 12 months, or the use of immunosuppressive medications, such as mycophenolate, antimetabolites, or mTOR inhibitors [5,31]. This implies that better and perhaps more frequent vaccination strategies would be needed for especially vulnerable populations [5], and further emphasizes that candidates for kidney transplant must be vaccinated before transplantation as much as possible [14].

The results of this systematic review should be considered according to their limitations. First, a meta-analysis could not be conducted because of the heterogeneity of the settings and follow-ups among the studies. Second, the number of studies was relatively small and included small sample sizes and heterogeneous populations. Third, there was no control or matching by age or sex in any study. Most end-stage kidney disease patients were in their late mid-age to the elderly range, whereas healthy individuals were younger and varied in age more than the patients. As the immunogenicity of younger people is higher than that of older adults, this bias should be controlled in future studies. Further, no study provided data separated by sex; therefore, sex differences could not be analyzed. Finally, the studies were heterogeneous in data collection, clinical setting, and follow-ups. In fact, several factors that can condition immune responses were not properly controlled in most studies. Overall, current evidence on immune response long-COVID symptoms by SARS-CoV-2 variants should be considered with caution at this stage.

## 5. Conclusions

In this systematic review, the humoral response in hemodialysis patients post-SARS-CoV-2 mRNA vaccination was found to be significantly lower when compared with the general population. Potential risk factors such as older age, use of immunosuppressive therapy, and type of vaccine were identified. Preliminary evidence suggests that the Moderna-NIAID vaccine elicits a more effective immune response than the Pfizer-BioNTech vaccine. Personalized vaccination strategies adapted to patients undergoing hemodialysis, particularly those older than 65 years and in active use of immunosuppressive therapy, are seemingly needed. All these data were based on heterogeneous studies, which did not permit the conduct of a meta-analysis.

## Figures and Tables

**Table 1:** Database formulas using literature search.

Pubmed Search Formula
#1 “Hemodialysis” [All Fields] #2 “humoral response” [All Fields] #3 “COVID vaccine” [All Fields] OR “COVID-19 Vaccines” [Mesh] OR “SARS-CoV-2 vaccine” [All Fields] OR “BNT162b2” [All Fields] OR “BNT162 Vaccine” [Mesh] OR “Pfizer-BioNTech” [All Fields] OR “mRNA-1273” [All Fields] OR “2019-nCoV Vaccine mRNA-1273” [Mesh] OR “Moderna” [All Fields] #4 #1 AND #2 #5 #1 AND #3 #6 #1 AND #2 AND #3
MEDLINE/CINAHL (via EBSCO) Search Formula
#1 “Hemodialysis” #2 “humoral response” #3 “COVID vaccine” OR “SARS-CoV-2 vaccine” OR “BNT162b2” OR “Pfizer-BioNTech” OR “mRNA-1273” OR “Moderna” #4 #1 AND #2 #5 #1 AND #3 #6 #1 AND #2 AND #3

**Table 2:** Studies investigating the humoral response of hemodialysis patients post-COVID-19 vaccination with primary series.

Author	Vaccine	Country	Sample	Age	Assay	Findings	
						First Dose	Second Dose
Danthu et al. [12]	BNT162b2/Pfizer-BioNTech	France	n = 159 HDP: n = 78 KTR: n = 74 Controls: n = 7	Mean (SD) HDP: 73.5 (12.8) KTR: 64.8 (11.5) Control: 51.6 (6.8)	Baseline: Abbott Alinity SARS-CoV-2 IgG (Chicago, IL, USA) Post-vaccination: LIAISON SARS-CoV-2 TrimericS IgG (DiaSorin, Saluggia, Italy)	14 days after the first injection, Ab titers in control group were 14.75-fold higher than HDP. No positive antibody levels were detected in KTR patients.	8 days after the second dose, Ab titers in controls and HDP increased 18.34-fold, and 1.65-fold, respectively. Ab in controls was 163.94-fold higher than HDP 8 days after the second dose. 30 days after the second dose, Ab titer in controls and HDP increased 0.85-fold, and 41.82-fold, respectively, compared with 8 days after the second dose. Ab in controls was 3.35-fold higher than HDP 30 days after the second dose.
Van Praet et al. [13]	BNT162b2/Pfizer-BioNTech & mRNA-1273/Moderna-NIAID	Belgium	n = 618 HDP: n = 543 Pfizer-BioNTech: n = 322 Moderna-NIAID: n = 221 Controls: n = 75 Pfizer-BioNTech: n = 37 Moderna-NIAID: n = 38	Median (range) Pfizer-BioNTech: 76 (66–82) Moderna-NIAID: 75 (65–82)	AdviseDx SARS-CoV-2 IgG II chemiluminescent microparticle immunoassay (Abbott, Ireland)	28 days after the first BNT162b2 dose, antibody titers in controls were 22.3-fold higher than in HDP. 56 days after the first BNT162b2 dose, antibody titers in controls were 5.2-fold higher than in HDP. 35 days after the first mRNA-1273 dose, antibody titers in controls were 14.8-fold higher than in HDP 63 days after the first mRNA-1273 dose, antibody titers in controls were 4.7-fold higher than in HDP.	ND
Sanders et al. [14]	mRNA-1273/Moderna-NIAID	Netherlands	n = 800 Controls: n = 191 CKD G4/5: n = 162 HDP: n = 159 KTR: n = 288	Mean (SD) Controls: 58.5 (13.0) CKD G4/5: 60.6 (13.4) HDP: 59.8 (14.3) KTR: 56.1 (14.0)	Validated fluorescent bead-based multiplex-immunoassay with a specificity and sensitivity of 99.7% and 91.6%	28 days after the first dose, antibody titers in responders were 1.8-fold higher in controls than in CKD stage 4 or 5 (CKD 4/5) patients, 4-fold higher than in HDP and 500-fold higher than in KTR.	28 days after the second dose, antibody titers in responders were 1.3-fold higher in controls than in CKD stage 4 or 5 (CKD 4/5) patients, 1.9-fold higher than in HDP and 127.4-fold higher than in (KTR).
Bertrand et al. [15]	BNT162b2/Pfizer-BioNTech	France	n = 55 HDP: n = 10 KTR: n = 45	Mean (SD) HDP: 71.2 (16.4) KTR: 63.5 (16.3)	AdviseDx SARS-CoV-2 IgG II chemiluminescent microparticle immunoassay (Abbott, Ireland)	21 days after the first dose, only one HDP (11.1%) and one KTR (2.2%) showed anti–SARS-CoV-2 antibodies. Antibody titers in responders were 1.74-fold higher in KTR as compared with HDP.	30 days after the second dose, eight HDP (88.9%) and eight KTRs (17.8%) developed SARS-CoV-2 antibodies. HDP and KTR antibody titers increased 5.88-fold and 2.16-fold, respectively. HDP responders had a 1.57-fold higher response than KTR responders after the second dose.
Duarte et al. [17]	BNT162b2/Pfizer-BioNTech	Portugal	Total: n = 67 HDP: n = 25 PDP: n = 42	Mean (SD) HDP: 75.1 (11.7) PDP: 60.5 (10.7)	MAGLUMI^®^ SARS-CoV-2 S-RBD IgG chemiluminescence kit. (Snibe Diagnostic, China)	21 days after the first dose, IgG titers in PDP were 5.45-fold higher than HDP. HDP was weakly associated with non-response after the first dose when compared with PDP	21 days after the second dose, IgG titers in PDP and HDP increased by 31.33-fold and 66.47-fold, respectively, with titers in PDP being 2.59-fold higher than HDP.
Jahn et al. [18]	BNT162b2/Pfizer-BioNTech	Germany	Total: n = 88 HDP: n = 72 Controls: n = 16	HDP: 68 (37–90) Controls: 45 (39–65)	LIAISON^®^ SARS-CoV-2-TrimericS IgG chemiluminescent immunoassay (Diasorin S.p.A., Saluggia, Italy)	ND	13 days after the second dose for controls and 17 days after for HDP, antibody titers were 2.1-fold higher in controls compared with HDP.
Zitt et al. [19]	BNT162b2/Pfizer-BioNTech	Austria	Total: n = 50 Seroconversion: n = 21 No seroconversion: n = 29	Mean (SD) Seroconversion: 67.6 (16.1) No seroconversion: 71.2 (12.9)	LIAISON^®^ SARS-CoV-2-TrimericS IgG chemiluminescent immunoassay (Diasorin S.p.A., Saluggia, Italy)	Compared with the baseline of being seronegative, 25 days after first dose, antibody titer in hemodialysis patients was 56.7 BAU/mL	Compared with the baseline of being seronegative, 28 days after the second dose, antibody titer in hemodialysis was 1565.0 BAU/mL

HDP = hemodialysis patients, KTR = kidney transplant recipients, CKD G4/5 (eGFR < 30 mL/min/1.73 m^2^) = chronic kidney disease stage 4/5, PDP = peritoneal dialysis patients, Ab = antibody, ND = no data.

**Table 3:** Studies investigating the humoral response of hemodialysis patients post-COVID-19 vaccination with booster.

Author	Vaccine	Country	Sample	Age	Assay	Findings after Booster
Bensouna et al. [16]	BNT162b2/Pfizer-BioNTech	France	n = 69 HDP: n = 38 PDP: n = 31	Median (range) 68 (53–76)	Elecsys Anti-SARS-CoV-2 S1 (Roche Diagnostics, Boulogne-Billancourt, France)	At least 3 weeks after the booster dose, the Ab of HDP and PDP increased by 26.6-fold as compared with the second dose. Patients with a greater increase in anti-S1 Ab levels after the third dose had lower Ab levels after the second dose, and a longer time interval between the second and the third dose.
Wang et al. [20]	mRNA-1273/Moderna-NIAID	United States	n = 63 Vaccination cohort: n = 42 Infection cohort: n = 21	Vaccination cohort: 63 (42–82) Infection cohort: 62 (41–80)	GenScript SARS-CoV-2 Surrogate Virus Neutralization Test Kit (GenScript Biotech Corporation, Piscataway, NJ, USA)	Compared with levels prior to the third dose of mRNA 1273, nAb-WT increased 18-fold and nAb-Omicron increased 23-fold after 23 and 24 days from the third dose, respectively.

nAb = neutralizing antibody, WT = wild-type strain; HDP = hemodialysis patients, PDP = peritoneal dialysis patients.

**Table 4:** Methodological quality (Newcastle-Ottawa Scale—NOS) of studies included in the review.

Study	Selection				Comparability		Outcome			
	Representative of the Exposed Cohort	Selection of Nonexposed Cohort	Ascertainment of Exposure	Outcome of Interest	Main Factor	Additional Factor	Assessment of Outcomes	Sufficient Follow-Up	Adequacy of Follow-Up	Total Score
Zitt et al. [19]	★		★	★			★	★	★	6/9
Jahn et al. [18]	★		★	★			★	★	★	6/9
Duarte et al. [17]	★		★	★			★	★	★	6/9
Bertrand et al. [15]				★			★	★	★	4/9
Sanders et al. [14]	★	★	★	★			★	★	★	7/9
Danthu et al. [12]	★		★	★			★	★	★	6/9
Wang et al. [20]	★		★	★			★	★	★	6/9
Van Praet et al. [13]	★	★	★	★			★	★	★	7/9
Bensouna et al. [16]	★		★	★			★	★	★	6/9

## Data Availability

All data are presented in the text.
